# Laveran et le Prix Nobel de physiologie ou de médecine

**DOI:** 10.48327/mtsi.v3i1.2023.310

**Published:** 2023-02-08

**Authors:** Jean-Paul Boutin

**Affiliations:** SFMTSI Société francophone de médecine tropicale et santé internationale (ancienne SPE), Hôpital Pitié-Salpêtrière, Pavillon Laveran, 47-83 Boulevard de l'Hôpital, 75651 Paris cedex 13, France; Service de santé des armées, 60 boulevard du gal Martial Valin, 75509 Paris Cedex 15; GISPE Groupe d'intervention en santé publique et épidémiologie, 82 boulevard Tellène, 13008 Marseille, France; * Actes du Colloque – Centenaire de la mort d'Alphonse Laveran. 24 novembre 2022, Paris / Proceedings of the Conference – Centenary of the death of Alphonse Laveran. 24 November 2022, Paris

**Keywords:** Alphonse Laveran, Prix Nobel, Découverte de l'hématozoaire du paludisme, Institut Pasteur, France, Alphonse Laveran, Discovery of the malarial parasite, Nobel Prize, Pasteur Institute, France

## Abstract

Dans quelles conditions les premiers Prix Nobel furent-ils attribués? Les personnalités qui ont proposé Alphonse Laveran pour le prix dès 1901 sont rappelées, au nombre desquelles Ronald Ross lauréat en 1902. En 1907, Karl Axel Hampus Mörner propose Alphonse Laveran pour le prix. Or il s'agit du recteur en exercice du Karolinska Institutet et président du Comité Nobel et de l'Assemblée Nobel. L'année précédente l'Assemblée Nobel avait pour la première fois décerné le prix de physiologie ou de médecine à deux lauréats, Camillo Golgi et Santiago Ramón y Cajal, « en reconnaissance de leurs travaux sur la structure du système nerveux ». En 1907, les propositions nombreuses, répétées et simultanées d’Élie Metchnikoff et Paul Ehrlich ont probablement fait l'objet d'un important débat, finalement tranché au bénéfice d'Alphonse Laveran. Nous expliquons pourquoi nous pensons que le choix du président a prévalu. En 1908, alors que Laveran devient à son tour, et de droit, l'une des personnalités qui proposent des candidats au prix, ses principaux concurrents de 1907 seront couronnés simultanément « en reconnaissance de leur travail sur l'immunité ».

Le long délai entre la découverte de l'hématozoaire du paludisme (1880) et l'attribution du prix « en reconnaissance de ses travaux sur le rôle joué par les protozoaires dans l'apparition des maladies » est remis en perspective et illustre ce qui sera presque toujours l'usage, contraire à la règle voulue par Alfred Nobel, d'une attribution du prix dans l'année qui suit la découverte.

Le don que fit Alphonse Laveran, dès le 22 décembre 1907, à l'Institut Pasteur sur le montant de son prix s’élève à 100 000 francs, soit un peu plus de la moitié des 190 000 francs de la bourse reçue du Comité Nobel. Sa valeur en termes de pouvoir d'achat en euros 2021 est estimée à plus de 400 000 euros. L'usage qu'en fit l'Institut Pasteur ressort clairement des minutes de son Conseil d'administration en 1908, comme ayant été principalement consacré à l'aménagement et l’équipement du Laboratoire des maladies tropicales que Laveran appelait de ses vœux dans les immeubles récemment achetés rue Falguière; le don n'a pas servi à la construction d'immeubles nouveaux.

## Abstract (en)

This article recalls the conditions under which the first Nobel Prizes were awarded. The scientific personalities who nominated Alphonse Laveran for the prize from 1901 to 1907 are recalled, among them Ronald Ross, winner in 1902. In 1907, Karl Axel Hampus Mörner submitted Alphonse Laveran for the prize. He was then the rector of the Karolinska Institutet, as well as the chairman of the Nobel Committee and the Nobel Assembly, and had never before expressed an interest in one scientist more than another. The previous year, the Nobel Assembly had for the first time awarded the prize for physiology or medicine to two laureates, Camillo Golgi and Santiago Ramón y Cajal “in recognition of their work on the structure of the nervous system”. In 1907, the numerous, repeated and simultaneous nominations of Élie Metchnikoff and Paul Ehrlich were probably the subject of an important debate, which finally turned out in favour of Alphonse Laveran. We explain why we think the choice of the president prevailed. In 1908, when Laveran became one of the nominators of the prize, his main competitors of 1907 were finally crowned simultaneously “in recognition of their work on immunity”.

The long delay between the discovery of the malarial parasite (1880) and the award of the prize “in recognition of his work on the role played by protozoa in the appearance of diseases” is put into perspective and illustrates what will almost always be the practice, contrary to Alfred Nobel's rule of awarding the prize within a year after the discovery. The particular circumstances of the award of the prize in 1907 are described.

The donation that Alphonse Laveran made on December 22nd 1907 to the Pasteur Institute out of the amount of his prize was 100,000 francs, *i.e.* a little more than half of the 190,000 francs grant received from the Nobel Committee. Its value, in terms of purchasing power in euros 2021, is estimated at over 400,000 euros. The use made of it by the Pasteur Institute is clearly shown in the minutes of its Board of Directors in 1908, as having been mainly devoted to the fitting out and equipment of the Laboratory of Tropical Diseases that Laveran was calling for in the buildings recently purchased on Rue Falguière (Paris); the donation was not used for the construction of new buildings.

Dans quelles conditions le septième Prix Nobel de physiologie ou de médecine a-t-il été attribué à Alphonse Laveran, qu'estce que représentait la bourse attribuée au lauréat et quel usage en fit-il? Tels sont les aspects abordés dans ce texte, avec quelques certitudes mais aussi de simples hypothèses. En 1907, Alphonse Laveran reçoit seul le Prix Nobel de physiologie ou médecine, tandis qu'Albert A. Michelson reçoit celui de physique pour ses instruments d'optique de précision et les recherches spectroscopiques et métrologiques réalisées à l'aide de ces instruments; Eduard Buchner reçoit celui de chimie pour ses recherches et sa découverte de la fermentation acellulaire; Rudyard Kipling reçoit celui de littérature en considération du pouvoir d'observation, de l'originalité, de l'imagination, de la force des idées et du remarquable talent de narration qui caractérisent ses créations; enfin, le prix de la paix est partagé entre Ernesto Teodoro Moneta pour son action dans la presse et dans les conférences de paix, tant publiques que privées, en vue d'une entente entre la France et l'Italie, et Louis Renault pour son influence décisive sur le déroulement et les résultats des Conférences de La Haye et de Genève [11].

Dès la première année, en 1901, le Prix Nobel de la paix a été partagé entre plusieurs lauréats, partage qui apparaîtra en 1902 pour le prix de physique tandis que le prix de physiologie ou de médecine est partagé pour la première fois en 1906 entre Camillo Golgi et Santiago Ramón y Cajal pour leur travail sur la structure du système nerveux. La voie était donc ouverte à une pratique de partage du prix qui est aujourd'hui devenue la règle [11].

Le processus de qualification des candidats est défini dès 1900 [11]. Le droit de soumettre des propositions de lauréat pour le Prix Nobel de physiologie ou de médecine est défini dans les statuts de la Fondation Nobel. Les personnes habilitées à proposer des candidatures pour le prix de physiologie ou de médecine sont de droit les membres de l'Assemblée Nobel du Karolinska Institut et à Stockholm; les membres suédois et étrangers des sections de médecine et de biologie de l'Académie royale des sciences de Suède; les précédents lauréats du Prix Nobel de physiologie ou de médecine; tous les membres du Comité Nobel de Médecine même s'ils ne font pas partie d'une des listes précédentes; les titulaires de postes permanents de professeurs titulaires dans les facultés de médecine en Suède et les titulaires de postes similaires dans les facultés de médecine ou institutions similaires au Danemark, en Finlande, en Islande et en Norvège; les titulaires de postes similaires dans au moins 6 autres facultés de médecine d'universités du monde entier, sélectionnées par l'Assemblée Nobel afin de garantir une « répartition appropriée de la tâche » entre les différents pays; enfin des scientifiques que l'Assemblée Nobel jugera bon de contacter. Toutes ces personnalités (dans la suite de ce texte dites proposants) sont donc appelées à faire des propositions, terme préféré à l'anglicisme en vogue de nomination.

Les scientifiques éligibles pour le prix de physiologie ou de médecine sont donc ceux qui ont été suggérés par ces proposants qui ont tous reçu une invitation du Comité Nobel à soumettre des noms pour examen pour le prix du prochain millésime. Le recueil des propositions débute un an auparavant. Aucun proposant ne peut proposer sa propre candidature au prix [11].

Dans la campagne de recueil de propositions lancée fin 1906 pour l'attribution du prix en 1907, il y eut 94 propositions dont seulement 2 pour Alphonse Laveran, alors que Paul Ehrlich fut proposé cette année-là 14 fois et Élie Metchnikoff 11 fois [11]. On note au passage les propositions faites pour Angelo Celli, Battista Grassi, David Bruce, Émile Roux, Carlos Finlay, Albert Calmette, Santiago Ramón y Cajal, Emil Kocher, Charles Richet, etc.

À partir de 1901, année après année Laveran a été proposé 13 fois, et même pour l'anecdote une fois encore pour le prix de 1908 (Tableau I). Parmi les proposants de Laveran au fil des années, on relève Raphaël Blanchard, l’éminent parasitologue et entomologiste qui avait été élu très jeune, à 37 ans, à l'Académie nationale de médecine en 1894, Laveran ayant été le rapporteur de son dossier de candidature, mais aussi Étienne-Jules Marey le physiologiste du mouvement qui le proposa 3 fois, ou encore Ronald Ross lauréat en 1902 pour ses travaux sur le paludisme [11].

**Tableau I:** Personnalités ayant proposé Alphonse Laveran pour le Prix Nobel de physiologie ou de médecine entre 1901 et 1908 Personalities who nominated Alphonse Laveran for the Nobel Prize in Physiology or Medicine between 1901 and 1908

Année	Nom du proposant	Pays
1901	Raphaël Blanchard	France
1901	Étienne-Jules Marey	France
1902	Étienne-Jules Marey	France
1902	Charles Firket	Belgique
1903	Knud Faber	Danemark
1903	Étienne-Jules Marey	France
1903	Raphaël Blanchard	France
1904	Sv Skvortzow	Pologne
1905	Paul Vuillemin	France
1906	Ronald Ross	Royaume-Uni
1906	A. Dilbert	France
1907	Karl A. H. Mörner	Suède
1907	Giulio Vassale	Italie
1908	Constant Houlbert	France

Les deux scientifiques qui proposent Laveran en 1907 sont Karl Axel Hampus Mörner (Fig. 1) et Giulio Vassale. Si tout le monde se souvient de Vassale, le père de l'endocrinologie, découvreur des parathyroïdes, ce n'est probablement pas lui le plus important dans l’élection de Laveran en 1907. Arrêtons-nous un peu sur la personne de K. A. H. Mörner (1854-1917). Professeur titulaire de chimie à l'Institut Karolinska, il a développé une méthode pour déterminer l'urée (test de Heller), et découvert en 1897 par analyse spectroscopique la myoglobine [2]. Il est le recteur de l'Institut Karolinska depuis 1898, date à laquelle la réflexion sur les règles d'attribution du prix Nobel de physiologie ou de médecine était d'actualité, le testament d'Alfred Nobel datant de 1895 et son décès de 1896. En tant que recteur il a participé aux longues négociations menées entre les héritiers d'Alfred Nobel, les exécuteurs testamentaires et les répartiteurs de prix pour rédiger les constitutions de la Fondation Nobel qui aboutissent en 1900. Mörner a été de 1901 jusqu’à sa mort président du Comité Nobel de l'Institut Karolinska, celui qui sollicite et recueille chaque année les propositions de lauréats. Il a laissé sa marque sur les règlements et les pratiques qui sont suivis lors de la sélection des lauréats et de la remise du prix [2]. En tout, il n'a fait que 4 propositions pour le prix, pour 2 personnes seulement: en 1907 pour Alphonse Laveran, puis par 3 fois de 1908 à 1910 pour Emil Fischer [11].

**Figure 1:**
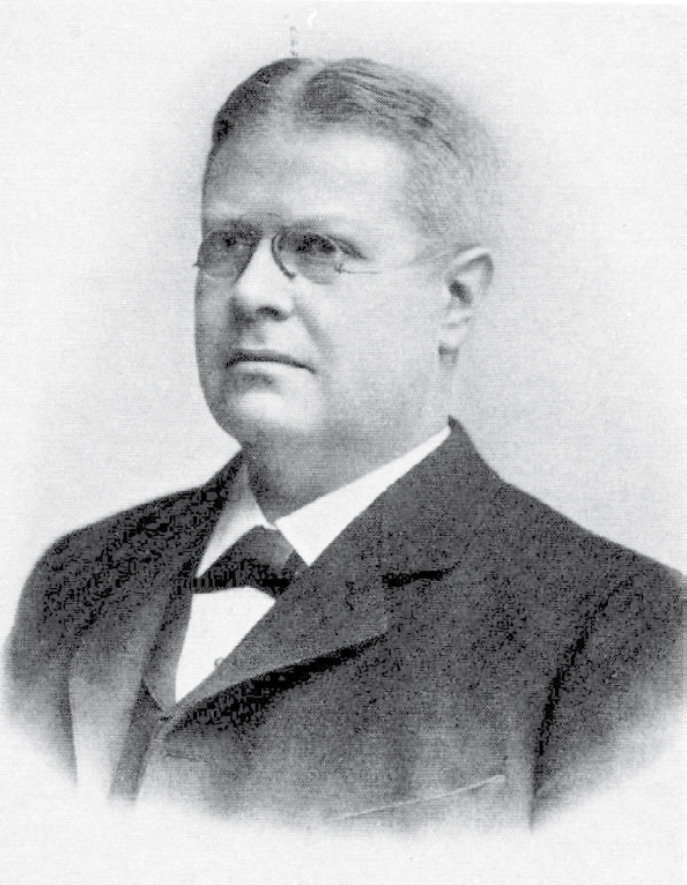
Karl Axel Hampus Mörner (1854-1917), recteur de l'Institut Karolinska à partir de 1898, président du Comité Nobel et de l'Assemblée Nobel de 1900 à 1917 (avant 1917, auteur inconnu [2]) Karl Axel Hampus Mörner (1854-1917), rector of the Karolinska Institute from 1898, president of the Nobel Committee and Nobel Assembly from 1900 to 1917 (before 1917, author unknown [2])

Le travail du Comité Nobel en amont n'est pas celui de l'Assemblée Nobel en aval, laquelle vote et désigne finalement le lauréat. Les archives de l'Assemblée sont secrètes, seules celles du Comité sont accessibles. On ne connaît donc pas la teneur des débats qui ont précédé le vote, ni la répartition des voix. Mais on reste étonné que les noms d'Ehrlich et Metchnikoff, tous deux géants de l'immunologie, proposés 40 fois depuis 1901 pour le premier et 53 fois pour le second, n'aient pas été retenus en 1907, alors que dès l'année 1906 l'attribution de ce prix à plusieurs lauréats était devenue réalité. Plusieurs hypothèses peuvent être émises: la persistance de doutes chez les électeurs sur la portée et la durabilité des découvertes de ces deux savants; le refus de la facilité en ne départageant pas des concurrents; le nationalisme des proposants (il y avait 7 proposants français ou russes pour Metchnikoff sur 11, et 5 allemands pour Ehrlich sur 14); et bien sûr l'importance de l'avis et de l'argumentaire du recteur Mörner qui, présidant le débat, était le mieux placé pour pousser Laveran qu'il avait proposé alors même qu'il était très avare de ses soutiens, 1907 étant la première fois où il fit une proposition. Finalement, Laveran est lauréat en 1907 et ses deux principaux concurrents seront co-lauréats en 1908 avec encore 19 et 13 propositions respectives [11]. En 1908, Alphonse Laveran devint à son tour et de droit, l'une des personnalités amenées à proposer des candidats au prix. Il fit entre 1908 et 1922, année de sa mort, 16 propositions dont à 5 reprises pour Aristides Agramonte y Simoni, à 4 reprises pour David Bruce et Carlos Finlay, 2 fois pour Émile Roux et une pour Auguste Trillat. À noter qu'il ne fit pas de proposition en 1911, 1916 et 1920 et que le prix ne fut pas remis entre 1915 et 1918 alors que le travail préparatoire avait été réalisé [11].

Le long délai entre la découverte de l'hématozoaire du paludisme (1880) et l'attribution du prix est en contradiction avec la volonté testamentaire d'Alfred Nobel de récompenser un travail fait dans l'année écoulée [11]. Cette règle n'a jamais été suivie. De l'aveu même de l'Assemblée Nobel, cela est dû premièrement à ce que les découvertes doivent être rendues publiques par écrit, ce qui prend du temps, et ensuite elles doivent être vérifiées par d'autres chercheurs avant d’être généralement adoptées par la communauté internationale. Pour résoudre ce problème, les jurés ont toujours été pragmatiques et ont interprété l'expression « année précédente » comme signifiant que le bénéfice de la découverte est devenu évident pour l'institution au cours de l'année précédente. Enfin, dans le cas des tout premiers lauréats il convenait aussi de prendre en compte la nouveauté du Prix par rapport à l'histoire de leur découverte sans pour autant tomber dans le risque d'un prix récompensant une carrière, ce qui était expressément contre-indiqué par Alfred Nobel. Pour ménager la règle et l'usage, l'Assemblée Nobel ne couronne pas Alphonse Laveran pour la découverte de l'hématozoaire du paludisme mais « en reconnaissance de ses travaux sur le rôle joué par les protozoaires dans l'apparition des maladies » [11] ce qui prend en compte non seulement la découverte du plasmodium mais aussi ses nombreuses avancées, le plus souvent en collaboration avec Félix Mesnil, sur les trypanosomes, les leishmanies, les coccidies, les piroplasmes, les hémogrégarines, etc. [8]. C'est d'ailleurs bien eu égard au rôle de père de la protozoologie médicale à partir de sa découverte de l'hématozoaire du paludisme que Laveran est distingué, comme le traduit clairement le discours de réception prévu par le Professeur Carl Sundberg (lequel avait proposé Carlos Finlay pour le prix cette année-là) au nom de l'Assemblée Nobel. Dans son discours inaugural, Sundberg rappelle combien il fut difficile dans les années 1880, en pleine diffusion de la pensée pastorienne définissant les bactéries comme causes de maladies infectieuses, de défendre l'idée de l’étiologie parasitaire à partir du cas du paludisme [10].

La cérémonie de remise du Prix était fixée au 10 décembre 1907, mais le décès deux jours plus tôt du roi Oscar II entraîna son annulation. Le discours préparé par Carl Sundberg ne fut pas prononcé, mais le texte en a été publié et reste disponible [10].

Signe des temps, le Prix Nobel reçu en 1907 par Alphonse Laveran était déjà le quatrième, en 7 ans, consacré à l'infectiologie, alors qu'il n'y en eut que 13 au cours du xx^e^ siècle récompensant des découvertes dans ce domaine: Emil von Behring (1901), Ronald Ross (1902), Robert Koch (1905), Alphonse Laveran (1907), Charles Nicolle (1928), Gerhard Domagk (1939), Alexandre Fleming, Ernst B. Chain et Howard Florey (1945), Paul Müller (1948), Max Theiler (1951), Selman A. Waksman (1952), John Enders, Thomas Weller et Frederick Robbins (1954), Baruch Blumberg et Daniel C. Gajdusek (1976), Stanley Prusiner (1997) [6]. Alphonse Laveran fut informé qu'il recevait le Prix Nobel par un courrier manuscrit en français de K. A. H. Mörner daté du 31 octobre 1907 (Fig. 2) indiquant que le prix serait décerné le 10 décembre suivant à Stockholm et que le montant est d'environ 190 000 francs; s'ensuivent des considérations pratiques sur la réception et le discours qu'il est invité à prononcer. On note que la présentation d'images pendant l'exposé se fait alors au moyen d'une projection scioptique à partir de plaques dont les dimensions doivent être précisées par avance.

**Figure 2:**
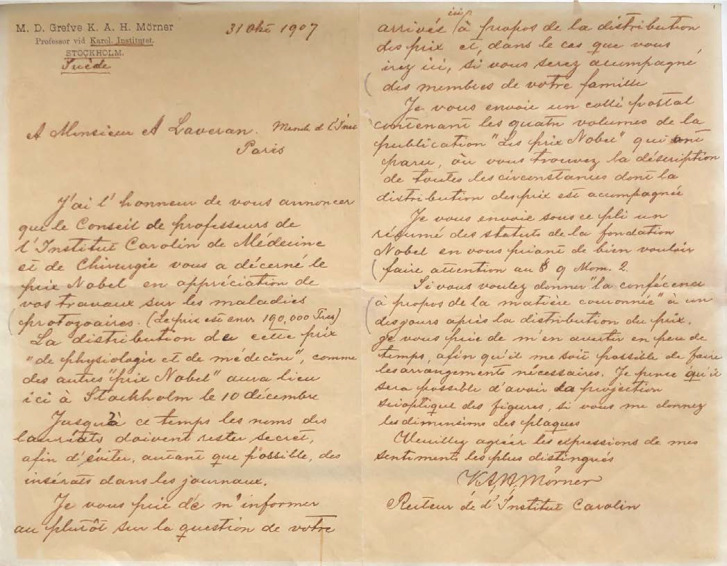
Lettre de Karl A. H. Mörner à Alphonse Laveran datée du 31 octobre 1907 (crédit photo: Musée du Service de santé des armées, Val-de-Grâce, Paris) Letter from Karl A. H. Mörner to Alphonse Laveran dated October 31^s1^ 1907 (photo credit: Musée du Service de santé des armées, Val-de-Grâce, Paris)

Si Mörner ne donne pas une valeur exacte de la bourse c'est qu'il faut prendre en compte les fluctuations du taux de change entre la couronne suédoise (SEK) et le franc français. La bourse à répartir en 1907 est de 138 796 SEK. Soit pour chacun des cinq prix la somme de 27 750 SEK. Laveran étant seul lauréat du Prix de physiologie ou de médecine, il reçoit cette somme correspondant donc à environ 190 000 francs.

À peine rentré de Stockholm, Alphonse Laveran adresse un courrier daté du 22 décembre 1907 à Émile Roux, directeur de l'Institut Pasteur, dans lequel il lui annonce sa décision de faire, grâce à la bourse du Prix Nobel, un don de 100 000 francs à la « maison de Pasteur ». Il ajoute qu'il désire que cette somme « soit employée à l'installation ou à l'entretien d'un petit laboratoire destiné spécialement à l’étude des protozoaires pathogènes » (Fig. 3) [1, 7, 8]. Pour se faire une idée de l'importance de cette somme, en euros actuels, l'Institut national de la statistique et des études économiques (INSEE) propose un convertisseur mesurant l’érosion monétaire qui permet d'exprimer, sur la période 1901-2021, le pouvoir d'achat d'une somme en euros ou en francs d'une année donnée en une somme équivalente d'une autre année, corrigée de l'inflation observée entre les deux années. Ainsi le pouvoir d'achat de 100 000 anciens francs de 1907 serait celui de 405 705 euros en 2021 [3].

**Figure 3:**
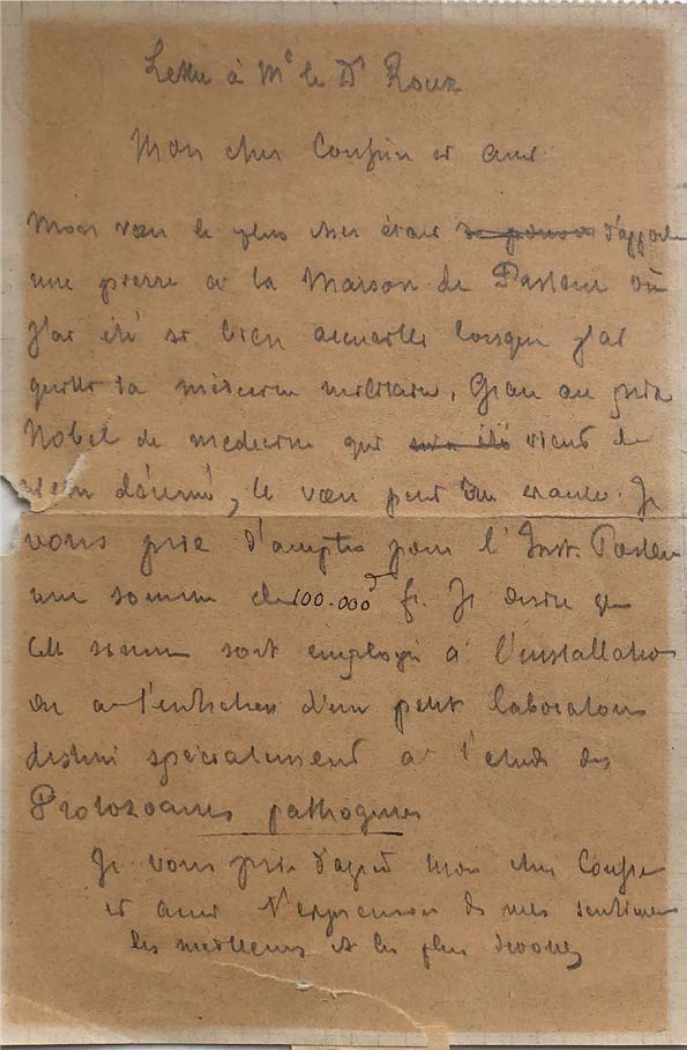
Projet de lettre de don d'Alphonse Laveran à l'Institut Pasteur (crédit photo: Musée du Service de santé des armées, Val-de-Grâce, Paris) Draft letter of donation from Alphonse Laveran to the Pasteur Institute (photo credit: Musée du Service de santé des armées, Val-de-Grâce, Paris)

Sur autorisation du Conseil d'administration de l'Institut Pasteur, cette somme fut placée par Émile Roux en titres dont seul le produit serait ensuite utilisé [5]. À la même période l'Institut finalisait le projet déjà ancien, grâce aux fonds de la succession Osiris, d'achat de l'immeuble et du terrain du 96 de la rue Falguière à Paris, appartenant à la Compagnie de Voitures « L'Urbaine », et contigus aux immeubles les plus récents de l'Institut [4]. Grâce à une partie du don de Laveran, l'immeuble, ou plutôt la maison d'habitation de la rue Falguière fut rapidement transformé « de façon à y installer des laboratoires; le deuxième étage fut mis à la disposition de Laveran qui, en raison de l'encombrement des laboratoires anciens, avait dû se contenter jusque-là d'une modeste chambre de travailleur. Le nouveau laboratoire comprenait, outre les pièces destinées au chef de service, au chef de laboratoire et aux assistants, des annexes pour les animaux: écuries, chenil, volière et aquariums; il prit le titre de Laboratoire des Maladies tropicales. » [8, 9] tandis que l'immeuble devint le Pavillon de pathologie exotique.

C'est dans son appartement-laboratoire du deuxième étage, dont la porte était indiquée par une modeste carte de visite fixée par une punaise, qu'Alphonse Laveran poursuivit ses recherches et reçut des chercheurs du monde entier jusqu'en 1922 (Fig. 4) [8, 9, 12].

**Figure 4:**
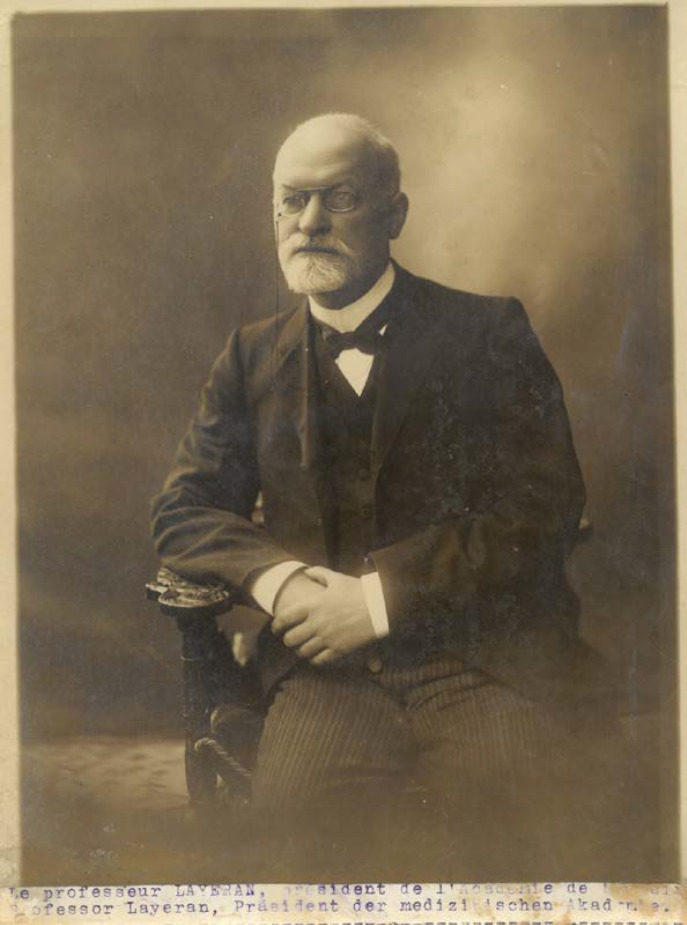
Alphonse Laveran, probablement après 1920 (crédit photo: J.-M. Milleliri) Alphonse Laveran, probably after 1920 (photo credit: J.-M. Milleliri)

## Liens Intérêts

L'auteur ne déclare aucun lien d'intérêt.
